# Prevalence of commonly diagnosed disorders in UK dogs under primary veterinary care: results and applications

**DOI:** 10.1186/s12917-021-02775-3

**Published:** 2021-02-17

**Authors:** Dan G. O’Neill, Hannah James, Dave C. Brodbelt, David B. Church, Camilla Pegram

**Affiliations:** 1 Pathobiology and Population Sciences, The Royal Veterinary College, Hawkshead Lane, North Mymms, AL9 7TA Hatfield, Herts UK; 2 The Kennel Club, Clarges Street, W1J 8AB Mayfair, UK; 3 Clinical Sciences and Services, The Royal Veterinary College, Hawkshead Lane, North Mymms, AL9 7TA Hatfield, Herts UK

**Keywords:** VetCompass, Electronic patient record, EPR, Breed, Dog, Epidemiology, Primary‐care, Veterinary, Pedigree, Purebred

## Abstract

**Background:**

Although dogs are a commonly owned companion animal in the UK, the species experiences many health problems that are predictable from demographic information. This study aimed to use anonymised veterinary clinical data from the VetCompass™ Programme to report the frequency of common disorders of dogs under primary veterinary care in the UK during 2016 and to explore effects associated with age, sex and neuter status.

**Results:**

From an available population of 905,543 dogs under veterinary care at 886 veterinary clinics during 2016, the current study included a random sample of 22,333 (2.47 %) dogs from 784 clinics. Prevalence for each disorder was calculated at the most refined level of diagnostic certainty (precise-level precision) and after grouping to a more general level of diagnostic precision (grouped-level precision). The most prevalent precise-level precision disorders recorded were periodontal disease (prevalence 12.52 %, 95 % CI: 12.09–12.97), otitis externa (7.30 %, 95 % CI: 6.97–7.65) and obesity (7.07 %, 95 % CI: 6.74–7.42). The most prevalent grouped-level disorders were dental disorder (14.10 %, 95 % CI: 13.64–14.56), skin disorder (12.58 %, 95 % CI: 12.15–13.02) and enteropathy (10.43 %, 95 % CI: 10.04–10.84). Associations were identified for many common disorders with age, sex and neuter.

**Conclusions:**

The overall findings can assist veterinarians and owners to prioritise preventive care and to understand demographic risk factors in order to facilitate earlier diagnosis of common disorders in dogs. The information on associations with age, sex and neuter status provides additional contextual background to the complexity of disorder occurrence and supports targeted health controls for demographic subsets of dogs.

## Background

Dogs are a common companion animal species in the UK, with 26 % of the UK adult population owning a dog and an estimated 9.9 million dogs owned in the UK [1]. Dog ownership has many reported benefits for both the humans and the dogs involved [2–5]. However, a growing body of evidence suggests that dogs experience many health problems that are not random events but may be associated with various risk factors including age, sex, neuter status and breed [6–8]. Whilst progress towards improved dog health and welfare requires collaboration between all those working in dog health, science and welfare [6, 9], the generation of reliable evidence on the breadth of health conditions across the wider dog population is a recurring and key constraint that limits effective welfare reforms and improvement [10].

Access to large data resources holding both demographic and health information on the general population of dogs is critical to provide reliable disorder prevalence information on dogs [11, 12]. National projects that hold anonymised veterinary clinical records from a diversity of primary-care practices have been identified as key resources for high quality health information relating to the wider population of dogs [10, 11, 13]. Over the past decade, several epidemiological projects housing large health databases on companion animals have been established including VetCompass (VetCompass 2020) and SAVSNET (SAVSNET 2019) in the UK, PETscan in the Netherlands (PETscan 2019), BARK in the US [14] and VetCompass Australia in Australia (VetCompass 2020). There are also plans to develop similar projects in other countries including New Zealand [15]. Epidemiological analyses of primary care veterinary data offer many advantages, including access to large volumes of clinical data that are contemporaneously recorded at the time of the clinical events by veterinary professionals and where diagnoses are updated over time as new information comes available [13]. The pace of publication of research based on these primary care clinical data is currently accelerating [16–19] and is contributing substantially to improved clinical practice activities [20, 21] and breed health reforms [22, 23].

An early study published in 2014 used primary-care clinical data to provide information on the overall disorder burden in dogs by reporting the prevalence of the 20 most common disorders recorded in dogs in England [7]. That paper placed particular focus on the effect of breed as a risk factor for common disorders and highlighted wide prevalence variation between breeds for common disorders. That study included 3,884 dogs from 89 clinics and identified the most frequently recorded disorders as otitis externa, periodontal disease and anal sac impaction. Following that original report on dogs overall, subsequent publications have reported the most common disorders within individual dog breeds and highlighted clearly differing disorder profiles between breeds: Border Terrier [24], Bulldog [25], Cavalier King Charles Spaniel [26], Chihuahua [27], French Bulldog [28], German Shepherd Dog [29], Greyhound [30], Labrador Retriever [31], Miniature Schnauzer [32], Pug [33], Rottweiler [34] and West Highland White Terrier [35]. These breed-specific studies also began to explore disorder associations with age, sex and neuter, and exposed the substantial complexity behind disorder occurrence in dogs. Identification of age, sex and neuter strata with higher risk for disorder occurrence suggests that health and welfare strategies to mitigate welfare harms from individual disorders may additionally benefit from targeted focus on predisposed age, sex or neuter strata to optimise outcomes [35]. There are currently aspirations to move towards greater targeting of welfare approaches using broad evidence bases that consider disorder prevalence, severity and duration along with other factors including predisposition, amenability to change and owner relevance [9, 36, 37]. To date, however, the applications of such targeted welfare strategies have been constrained by limited availability of published evidence on disorder risk within age, sex and neuter strata [16, 38].

Association between age and disorder occurrence was reported in a recent study of dogs presenting to veterinary clinics in the Republic of Korea that showed distinct disorder profiles across age groups [39]. Young dogs (< 1 year) had higher risk of presenting with diarrhoea, vomiting and infectious diseases, whilst older dogs (> 10 years) were more likely to present with disorders such as heart disease, kidney disease, Cushing’s disease, and mammary tumours. Substantial variation in disorder risks between age strata was also reported using questionnaire health datactions to address these concerns [a collected on 43,005 dogs registered with the Kennel Club (KC) in the UK [40]. Other studies of specific disorders have also highlighted age, sex and neuter status as key risk factors to consider during evaluations for disorder occurrence [16, 38, 41–43].

An enhanced evidence base on the overall disorder burden of dogs broken down by breed, age and sex could support the development of targeted health strategies for dogs by a range of stakeholders. For example, the UK KC has designed its breed health strategies to prioritise key health concerns in order to achieve maximum health improvement overall [44]. Within this overall plan, the KC implemented its ‘Breed Health and Conservation Plans’ programme in 2016 to collate data from mutliple sources and provide breeds with an evidence-based overview of current health concerns within their population as well as providing a series of useful recommended actions to address these concerns [37]. However, the limited evidence base that was available on the frequency and risk factors for disorder occurrence in the earlier days of these Breed Health and Conservation Plans was a critical limitation to their utility at that time, particularly in respect of prioritisation of health concerns. Increased information on disorder frequency along with age, sex and breed risk effects would strengthen the Breed Health and Conservation Plans, as well as many other health strategies, to optimise delegation of resources in a targeted fashion and improve aspects such as owner awareness, assisting veterinarians working in practice and funding of further research in under-investigated areas [10].

With a perspective of providing reliable information on common disorders in the wider population of dogs, this study aimed to use anonymised veterinary clinical data from the VetCompass™ Programme (VetCompass 2020) to report the frequencies of common disorders of dogs under primary veterinary care in the UK during 2016. Prevalence was calculated at the most refined level of diagnostic certainty (precise-level precision) for each disorder and also following grouping to a more general level of diagnostic precision (grouped-level precision). Given the value of deeper understanding of specific risk factors for disease, the study placed special focus on exploring effects associated with age, sex and neuter status. These results could assist veterinary practitioners, breeders and owners with an evidence base to understand and predict likely disorder occurrence and to identify key health and welfare opportunities for dogs. The overall results reported from this study could also act as a benchmark baseline for wider comparison in other studies that elect to focus on specific breeds, ages, sexes or neuter status.

## Results

### Demography

From an available population of 905,543 dogs under veterinary care at 886 veterinary clinics during 2016, the current study included a random sample of 22,333 (2.47 %) dogs from 784 clinics. The median age of this sample of dogs was 4.40 years (interquartile range [IQR] 1.87–8.05, range 0.01–20.46). Of sample dogs with information available, there were 10,540 (47.35 %) females and 10,097 (45.36 %) neutered animals. The median age of females (4.46 years, IQR 1.93–8.11, range 0.01–20.46) did not differ significantly to males (4.34 years, IQR 1.84–7.99, range 0.01–19.54) (*P* = 0.087). The median age of entire animals (2.79 years, IQR 1.20–6.40, range 0.01–19.83) was significantly younger than for neutered animals (6.12 years, IQR 3.58–9.28, range 0.17–20.46) (*P* < 0.001). Females were more likely to be neutered (4,856/10,540, 46.07 %) than males (5,241/11,718, 44.73 %) (*P* = 0.044). Data completeness for each variable was: sex 99.7 %, neuter 99.7 % and age 98.8 %.

### Summary disorder occurrence

**Fig. 1:**
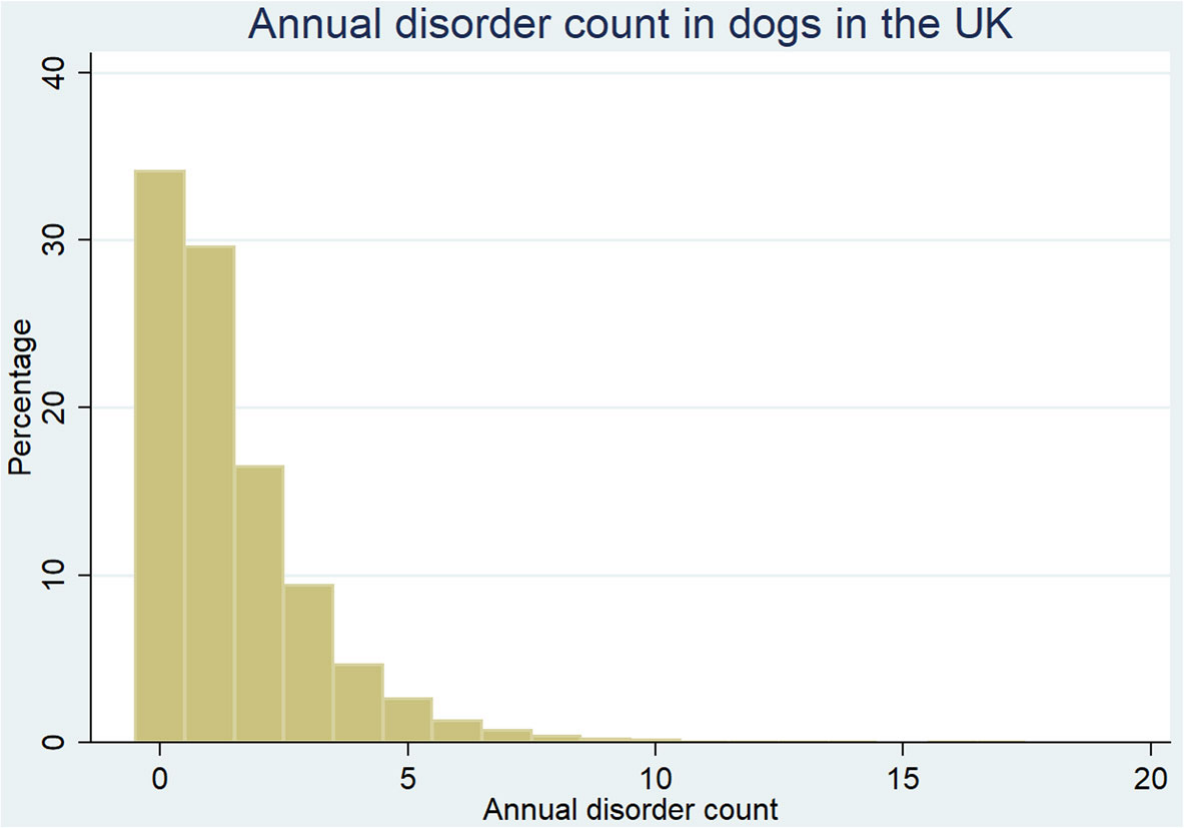
Annual disorder count for dogs (*n* = 22,333) under UK primary veterinary care from January 1st 2016 to December 31st, 2016 at practices participating in the VetCompass™ Programme

From the random sample of 22,333 dogs whose EPRs were manually examined to extract all recorded disorder data for 2016, there were 14,704 (65.84 %) dogs with at least one disorder recorded during 2016. The EPRs of the remaining 7,629 (34.16 %) dogs had no disorder recorded and either presented for prophylactic management only or did not present at all during 2016. The median annual disorder count per dog during 2016 was 1 disorder (IQR 0–2, range 0–17) (Fig. 1).

The proportion of females (65.51 %) with at least one disorder recorded did not differ to males (66.31 %) (*P* = 0.211). Neutered animals (71.66 %) had a higher probability of having at least one disorder recorded that entire animals (61.17 %) (*P* < 0.001). There was some evidence that the median annual disorder count was higher in males (1, IQR 0–2, range 0–14) than in females (1, IQR 0–1, range 0–17) (*P* = 0.049). The median age of dogs with at least one disorder recorded (5.15 years, IQR 2.24–8.84, range 0.16–20.46) was older than for dogs that did not have any disorder recorded (3.33 years, IQR 1.42–6.28, 0.01–19.54) (*P* < 0.001). The median annual disorder count was higher in neutered (1, IQR 0–2, range 0–17) than in entire (1, IQR 0–2, range 0–16) (*P* < 0.001). There was a positive correlation between age and disorder count (Spearman’s rho 0.24, *P* < 0.001).

### Precise‐level disorder occurrence

The study included 32,243 unique disorder events recorded during 2016 that encompassed 678 precise-level disorder terms. The most prevalent precise-level precision disorders recorded were periodontal disease (*n* = 2,797, prevalence 12.52 %, 95 % CI: 12.09–12.97), otitis externa (1,631, 7.30 %, 95 % CI: 6.97–7.65), obesity (1,580, 7.07 %, 95 % CI: 6.74–7.42), overgrown nail(s) (1,233, 5.52 %, 95 % CI: 5.23–5.83), anal sac impaction (1,071, 4.80 %, 95 % CI 4.52–5.08) and diarrhoea (852, 3.81 %, 95 % CI 3.57–4.07) (Table 1).

**Table 1:** Prevalence of the 70 most common disorders (at least 100 events) at a precise-level of diagnostic precision recorded in dogs (*n* = 22,333) under primary-care veterinary care at UK practices participating in the VetCompass™ Programme from January 1st to December 31st, 2016. The odds ratio and *P*-values reflect the odds in males compared to females, and the odds in neutered dogs compared to entire dogs. *P*-values less than 0.05 shown in bold. *CI confidence interval

Precise-level disorder term	No.	Overall %	95 % CI*	Odds ratio: male compared with female	95 % confidence interval	*P*-Value	Odds ratio: neutered compared with entire	95 % confidence interval	*P*-Value	Median age (years)
Periodontal disease	2797	12.52	12.09–12.97	1.01	0.92–1.11	0.903	1.22	1.11–1.34	**< 0.001**	7.54
Otitis externa	1631	7.30	6.97–7.65	1.23	1.09–1.39	**0.001**	0.99	0.87–1.13	0.881	4.72
Obesity	1580	7.07	6.74–7.42	0.89	0.79-1.00	0.055	1.44	1.27–1.64	**< 0.001**	5.99
Overgrown nail(s)	1233	5.52	5.23–5.83	0.81	0.70–0.93	**0.003**	0.91	0.79–1.06	0.229	4.68
Anal sac impaction	1071	4.80	4.52–5.08	0.96	0.83–1.11	0.572	1.15	0.99–1.34	0.069	5.24
Diarrhoea	852	3.81	3.57–4.07	1.04	0.87–1.23	0.667	1.04	0.87–1.25	0.671	2.93
Vomiting	679	3.04	2.82–3.27	0.97	0.80–1.17	0.749	0.99	0.81–1.21	0.944	3.54
Lameness	591	2.65	2.44–2.87	1.04	0.86–1.27	0.679	1.05	0.85–1.29	0.663	5.50
Osteoarthritis	522	2.34	2.14–2.54	1.24	1.01–1.52	**0.045**	1.33	1.06–1.67	**0.013**	11.30
Aggression	501	2.24	2.05–2.45	1.76	1.41–2.21	**< 0.001**	1.09	0.88–1.37	0.431	5.46
Conjunctivitis	500	2.24	2.05–2.44	1.00	0.81–1.23	0.979	1.04	0.83–1.30	0.739	4.03
Heart murmur	475	2.13	1.94–2.32	1.01	0.80–1.27	0.943	0.80	0.63–1.02	0.071	10.03
Skin mass	463	2.07	1.89–2.27	1.13	0.92–1.40	0.245	1.20	0.96–1.51	0.108	9.11
Flea infestation	458	2.05	1.87–2.25	0.89	0.71–1.12	0.320	0.71	0.56–0.91	**0.006**	4.18
Pruritus	363	1.63	1.46–1.80	0.94	0.74–1.20	0.617	1.03	0.80–1.33	0.826	4.79
Allergy	350	1.57	1.41–1.74	1.07	0.84–1.36	0.605	1.08	0.84–1.40	0.519	4.97
Undesirable behaviour	334	1.50	1.34–1.66	1.07	0.83–1.40	0.594	1.26	0.95–1.66	0.110	3.47
Pyoderma	325	1.46	1.30–1.62	0.96	0.75–1.24	0.757	0.85	0.65–1.10	0.209	5.37
Lipoma	322	1.44	1.29–1.61	1.22	0.94–1.58	0.127	1.58	1.18–2.11	**0.002**	10.10
Claw injury	309	1.38	1.23–1.55	1.08	0.84–1.38	0.558	1.03	0.79–1.33	0.851	4.89
Pododermatitis	303	1.36	1.21–1.52	1.18	0.91–1.53	0.210	1.29	0.98–1.69	0.070	5.58
Gastroenteritis	298	1.33	1.19–1.49	1.23	0.93–1.62	0.153	1.15	0.86–1.54	0.357	3.87
Foreign body	283	1.27	1.12–1.42	1.53	1.13–2.08	**0.006**	1.20	0.88–1.63	0.262	2.81
Post-operative wound complication	265	1.19	1.05–1.34	0.84	0.56–1.26	0.394	0.93	0.60–1.43	0.727	3.33
Atopic dermatitis	256	1.15	1.01–1.29	1.00	0.76–1.32	0.989	1.36	1.02–1.83	**0.040**	5.75
Wound	250	1.12	1.01–1.29	1.42	1.05–1.92	**0.022**	0.83	0.61–1.13	0.237	3.84
Skin cyst	246	1.10	0.97–1.25	0.84	0.63–1.12	0.244	0.89	0.66–1.21	0.466	7.63
Patellar luxation	233	1.04	0.91–1.19	0.85	0.64–1.14	0.289	1.07	0.79–1.47	0.655	4.29
Retained deciduous tooth	225	1.01	0.88–1.15	1.27	0.86–1.88	0.220	0.99	0.64–1.54	0.964	1.33
Coughing	220	0.99	0.86–1.12	1.68	1.21–2.33	0.002	0.93	0.67–1.30	0.685	6.02
Kennel Cough	215	0.96	0.84–1.10	1.13	0.82–1.56	0.462	0.91	0.65–1.29	0.602	3.78
Cataract	209	0.94	0.81–1.07	0.87	0.63–1.21	0.405	1.20	0.84–1.71	0.308	12.00
Umbilical hernia	208	0.93	0.81–1.07	0.76	0.48–1.18	0.225	0.94	0.56–1.59	0.831	1.03
Anal sac infection	200	0.90	0.78–1.03	0.79	0.58–1.08	0.137	0.78	0.57–1.07	0.123	5.96
Moist dermatitis	200	0.90	078-1.03	1.54	1.11–2.13	**0.010**	0.74	0.53–1.03	0.070	5.11
Alopecia	186	0.83	0.72–0.96	0.89	0.63–1.27	0.530	0.70	0.48–1.01	0.054	5.19
Haircoat disorder	183	0.82	0.71–0.95	1.14	0.80–1.63	0.480	0.90	0.62–1.31	0.586	3.66
Disorder unspecified	181	0.81	0.70–0.94	1.05	0.69–1.59	0.819	1.21	0.78–1.88	0.405	12.71
Thin	177	0.79	0.68–0.92	0.97	0.64–1.47	0.889	0.77	0.49–1.19	0.236	2.39
Dental disease	177	0.79	0.68–0.92	0.94	0.67–1.32	0.731	1.28	0.89–1.85	1.182	6.37
Urinary incontinence	175	0.78	0.67–0.91	0.24	0.16–0.37	**< 0.001**	1.90	1.23–2.95	**0.004**	11.60
Corneal ulceration	172	0.77	0.66–0.89	0.97	0.67–1.42	0.885	0.94	0.63–1.40	0.759	7.42
Dog bite	166	0.74	0.63–0.86	1.10	0.77–1.59	0.593	0.75	0.51–1.10	0.139	4.07
Ocular discharge	165	0.74	0.63–0.86	0.80	0.56–1.17	0.250	0.79	0.53–1.18	0.255	3.63
Laceration	160	0.72	0.61–0.84	1.50	1.02–2.20	**0.037**	1.51	1.01–2.57	**0.043**	3.58
Dermatitis	160	0.72	0.61–0.84	0.87	0.60–1.26	0.475	0.98	0.66–1.44	0.908	4.61
Collapsed	158	0.71	0.60–0.83	1.02	0.70–1.51	0.905	0.87	0.58–1.29	0.480	12.37
Skin lesions	156	0.70	0.59–0.82	1.18	0.79–1.77	0.411	0.88	0.58–1.34	0.562	3.22
Colitis	155	0.69	0.59–0.81	1.41	0.95–2.09	0.088	1.26	0.83–1.90	0.280	4.19
Seizure disorder	151	0.68	0.57–0.79	1.82	1.23–2.76	**0.003**	0.96	0.65–1.43	0.850	8.16
Tick infestation	151	0.68	0.57–0.79	0.97	0.66–1.42	0.870	0.99	0.66–1.49	0.973	3.84
Papilloma	150	0.67	0.57–0.79	1.01	0.70–1.46	0.947	1.16	0.79–1.71	0.453	10.63
Cruciate disease	149	0.67	0.56–0.78	0.71	0.50–1.01	0.055	1.34	0.91–1.97	0.138	7.57
Post-operative complication (not wound-related)	148	0.66	0.56–0.78	0.84	0.56–1.26	0.394	0.93	0.60–1.43	0.727	2.42
Anorexia	146	0.65	0.55–0.77	1.06	0.72–1.58	0.755	0.90	0.60–1.36	0.617	7.43
Stiffness	142	0.64	0.55–0.75	1.08	0.75–1.57	0.665	1.16	0.78–1.72	0.467	10.72
Urinary tract infection	131	0.59	0.49–0.70	0.33	0.21–0.50	**< 0.001**	1.85	1.17–2.92	**0.008**	8.50
Anxious/distressed	130	0.58	0.49–0.69	1.11	0.74–1.67	0.617	1.03	0.67–1.57	0.894	4.65
Cryptorchidism	127	0.57	0.47–0.68	~	~	~	~	~	~	1.34
Lethargy	126	0.56	0.47–0.67	1.20	0.78–1.85	0.410	1.13	0.72–1.77	0.609	5.21
Dietary indiscretion	125	0.56	0.47–0.67	1.27	0.81–1.98	0.291	1.12	0.70–1.79	0.637	2.04
Weight loss	120	0.54	0.45–0.64	0.78	0.51–1.18	0.240	0.64	0.41–0.99	**0.044**	11.20
Adverse reaction to drug	117	0.52	0.43–0.63	1.84	1.17–2.87	**0.008**	1.05	0.67–1.65	0.836	3.54
Musculoskeletal injury	115	0.51	0.43–0.62	1.40	0.91–2.14	0.127	1.34	0.85–1.12	0.207	4.74
Ear disorder	113	0.51	0.42–0.61	1.29	0.84–1.98	0.247	1.15	0.73–1.79	0.545	4.33
Polyuria/polydipsia	110	0.49	0.41–0.59	0.84	0.54–1.29	0.426	1.34	0.84–2.14	0.225	10.31
Flea bite hypersensitivity	108	0.48	0.40–0.58	0.87	0.55–1.37	0.547	0.64	0.40–1.02	0.061	4.85
Gastritis	105	0.47	0.38–0.57	0.78	0.47–1.28	0.328	0.88	0.52–1.48	0.622	4.05
Spinal pain	103	0.46	0.38–0.56	0.85	0.55–1.31	0.463	0.89	0.57–1.40	0.624	7.91
Mammary mass	102	0.46	0.37–0.55	0.01	0.00-0.07	**< 0.001**	0.41	0.25–0.69	**0.001**	10.58

Among the 70 most common precise-level disorders, the odds for 14 (20.0 %) disorders differed between the sexes after accounting for confounding. Males had higher odds than females for 10 disorders: otitis externa, aggression, coughing, seizure disorder, foreign body, adverse reaction to drug, moist dermatitis, wound, laceration, osteoarthritis. Females had higher odds than males for 4 disorders: urinary incontinence, urinary tract infection, mammary mass and overgrown nail(s). After accounting for confounding, there were 11/70 (15.7 %) precise-level disorders with differing odds between entire and neutered animals. Neutered animals had higher odds than entire animals for 8 disorders: obesity, periodontal disease, lipoma, urinary incontinence, urinary tract infection, osteoarthritis, atopic dermatitis, laceration. There were 3 disorders with higher odds in entire animals compared with neutered animals: mammary mass, flea infestation and weight loss. The median age of dogs recorded with each of the 70 most common precise-level disorders varied from 1.03 years for umbilical hernia to 12.71 years for disorder unspecified (Table 1).

### Grouped‐level disorder occurrence

There were 68 distinct grouped-level disorder terms recorded. The most prevalent grouped-level disorders were dental disorder (*n* = 3,148, prevalence: 14.10 %, 95 % CI: 13.64–14.56), skin disorder (2,810, 12.58 %, 95 % CI: 12.15–13.02), enteropathy (2,330, 10.43 %, 95 % CI: 10.04–10.84), musculoskeletal (1,929, 8.64 %, 95 % CI 8.27–9.01), ear disorder (1,825, 8.17 %, 7.82–8.54) and obesity (1,580, 7.07 %, 95 % CI: 6.74–7.42) (Table 2).

**Table 2:** Prevalence of the 36 most common disorders (at least 100 events) at a grouped-level of diagnostic precision recorded in dogs (*n* = 22,333) under primary-care veterinary care at UK practices participating in the VetCompass™ Programme from January 1st to December 31st, 2016. The odds ratio and *P*-values reflect the odds in males compared to females, and the odds in neutered dogs compared to entire dogs. *CI confidence interval

Grouped-level disorder term	No.	Overall %	95 % CI*	Odds ratio: male compared with female	95 % confidence interval	*P*-Value	Odds ratio: neutered compared with entire	95 % confidence interval	*P*-Value	Median age (years)
Dental disorder	3148	14.10	13.64–14.56	1.00	0.91–1.09	0.948	1.22	1.11–1.35	**< 0.001**	7.12
Skin disorder	2810	12.58	12.15–13.02	1.01	0.92–1.11	0.761	1.00	0.91–1.10	0.991	5.00
Enteropathy	2330	10.43	10.04–10.84	1.07	0.96–1.19	0.233	1.05	0.93–1.18	0.422	3.42
Musculoskeletal disorder	1929	8.64	8.27–9.01	1.18	1.05–1.32	**0.004**	1.19	1.06–1.34	**0.004**	7.53
Ear disorder	1825	8.17	7.82–8.54	1.21	1.07–1.35	**0.002**	1.03	0.92–1.17	0.584	4.82
Obesity	1580	7.07	6.74–7.42	0.89	0.79-1.00	0.052	1.44	1.27–1.64	**< 0.001**	5.99
Claw/nail disorder	1577	7.06	6.73–7.41	0.87	0.77–0.99	**0.032**	0.94	0.82–1.07	0.341	4.74
Ophthalmological disorder	1567	7.02	6.68–7.36	0.93	0.82–1.06	0.287	1.11	0.97–1.27	0.121	6.45
Anal sac disorder	1248	5.59	5.29–5.90	0.92	0.81–1.05	0.231	1.07	0.93–1.23	0.350	5.39
Mass	1169	5.23	4.95–5.53	0.95	0.82–1.09	0.437	1.02	0.88–1.18	0.760	9.19
Behaviour disorder	1140	5.10	4.82–5.40	1.24	1.07–1.43	**0.004**	1.19	1.03–1.39	**0.023**	4.65
Neoplasia	1140	5.10	4.82–5.40	1.03	0.89–1.19	0.679	1.11	0.96–1.29	0.172	9.19
Parasite infestation	850	3.81	3.56–4.07	0.93	0.78–1.11	0.422	0.77	0.64–0.93	**0.006**	3.14
Traumatic injury	822	3.68	3.44–3.94	1.33	1.12–1.58	**0.001**	1.01	0.84–1.20	0.946	3.68
Upper respiratory tract disorder	789	3.53	3.29–3.78	1.25	1.05–1.49	**0.012**	0.90	0.75–1.08	0.257	4.37
Heart disease	633	2.83	2.62–3.06	0.97	0.80–1.19	0.798	0.91	0.74–1.12	0.385	10.17
Complication associated with clinical care	417	1.87	1.69–2.05	0.82	0.65–1.04	0.097	0.89	0.70–1.14	0.372	3.09
Female reproductive disorder	328	1.47	1.32–1.64	~	~	~	0.37	0.28–0.50	**< 0.001**	3.77
Brain disorder	320	1.43	1.28–1.60	1.52	1.16–1.99	**0.002**	1.15	0.87–1.52	0.318	9.14
Underweight	316	1.41	1.26–1.58	0.93	0.70–1.24	0.625	0.63	0.48–0.90	**0.004**	6.51
Foreign body	283	1.27	1.12–1.42	1.53	1.13–2.08	**0.006**	1.20	0.87–1.63	0.262	2.81
Lethargy	273	1.22	1.08–1.38	0.90	0.63–1.20	0.475	0.80	0.59–1.08	0.143	5.65
Urinary system disorder	267	1.20	1.06–1.35	0.44	0.33–0.59	**< 0.001**	1.32	0.98–1.79	0.072	7.87
Hernia	255	1.14	1.01–1.29	0.80	0.53–1.17	0.247	0.85	0.55–1.31	0.458	1.09
Spinal cord disorder	216	0.97	0.84–1.10	1.09	0.79–1.51	0.587	1.06	0.75–1.49	0.740	9.56
Male reproductive system disorder	199	0.89	0.77–1.02	~	~	~	0.31	0.19–0.48	**< 0.001**	1.72
Incontinence	192	0.86	0.74–0.99	0.29	0.20–0.43	**< 0.001**	1.68	1.12–2.53	**0.013**	11.70
Endocrine system disorder	191	0.86	0.74–0.98	0.87	0.63–1.20	0.400	1.25	0.88–1.78	0.200	10.84
Disorder not diagnosed	181	0.81	0.70–0.94	1.03	0.68–1.56	0.880	1.18	0.76–1.83	0.464	12.71
Collapsed	164	0.73	0.63–0.86	1.06	0.73–1.55	0.750	0.89	0.60–1.32	0.547	12.35
Appetite disorder	156	0.70	0.59–0.82	1.03	0.70–1.51	0.889	1.00	0.67–1.50	0.996	7.43
Intoxication	154	0.69	0.59–0.81	1.44	0.93–2.22	0.099	0.99	0.63–1.54	0.956	2.36
Lower respiratory tract disorder	151	0.68	0.57–0.79	1.07	0.73–1.58	0.717	1.09	0.72–1.63	0.692	10.06
Adverse reaction to drug	151	0.68	0.57–0.79	1.55	1.05–2.29	**0.026**	1.15	0.77–1.72	0.496	3.06
Liver disorder	128	0.57	0.48–0.68	1.07	0.72–1.58	0.750	0.89	0.60–1.35	0.593	11.40
Polyuria/polydipsia	110	0.49	0.41–0.59	0.84	0.54–1.29	0.425	1.34	0.84–2.14	0.222	10.31

Among the 36 most common grouped-level disorders, the odds for 11 (30.5 %) disorders differed between the sexes after accounting for confounding. Males had higher odds than females for 8 disorders: traumatic injury, ear disorder, brain disorder, musculoskeletal disorder, behaviour disorder, foreign body, upper respiratory tract disorder, and adverse reaction to drug. Females had higher odds than males for 3 disorders: urinary system disorder, incontinence and claw/nail disorder. After accounting for confounding, there were 9/36 (25.0 %) grouped-level disorders with differing odds between entire and neutered animals. Neutered animals had higher odds than entire animals for 5 disorders: obesity, dental disorder, musculoskeletal disorder, incontinence and behaviour disorder. There were 4 disorders with higher odds in entire animals compared with neutered animals: female reproductive disorder, male reproductive system disorder, underweight and parasite infestation (Table 2).

## Discussion

 This is the largest study to date using primary-care veterinary data that reports on the common disorders in UK dogs. The study placed specific focus on exploring age, sex and neuter associations with disorder occurrence. At a precise level of diagnostic precision, the most commonly recorded disorders were periodontal disease, otitis externa, obesity, overgrown nail(s) and anal sac impaction. Whilst at a grouped level of diagnostic precision, the most common groups were dental disorder, skin disorder, enteropathy and musculoskeletal disorder. Awareness of the most common disorders of dogs can assist efforts to prioritise health reforms in dogs at a species level [45, 46]. Differing associations between categories of sex, neuter and age were reported for many common disorders, suggesting that these demographic features are important factors that need to be considered when exploring the epidemiology of these disorders and during the application of epidemiological information into health reforms. The overall findings can assist veterinarians and owners to prioritise preventive care and to facilitate earlier diagnosis of common disorders within dogs. The information on associations with age, sex and neuter provides additional contextual background to the complex world of disorder occurrence and can support targeted health controls for these subsets of dogs.

A smaller previous UK study using primary veterinary clinical data from 2009 to 2013 reported the most common disorders in dogs as otitis externa, periodontal disease, anal sac impaction, overgrown nail(s) and degenerative joint disease [7]. These findings are largely in line with the current study, though the current study reports a slightly higher prevalence of obesity and lower prevalence of osteoarthritis. Although this could indicate a genuine change in disorder prevalence over time, these differences are more likely to reflect methodological differences between the studies. Compared to the 3,998 dogs in the earlier report, the larger sample size of 22,333 dogs in the current study offers greater current precision. In addition, the methods used to extract disorder data from the clinical records have also advanced considerably during the intervening years [47], meaning that the current study was more highly powered and technologically enabled than the previous study. The ranking of the most common disorders in the current study that relied on veterinary clinical records differs substantially to results based on other data resources. Analyses of pet insurance records in Sweden have reported that skin and gastrointestinal disorders feature highly in dogs [48, 49]. A questionnaire-based survey of UK owners of dogs registered with the KC reported lipoma (4.3 %), skin cyst (3.1 %) and allergic skin disorder (2.7 %) as the most common disorders in dogs, possibly reflecting the prioritisation of the personal concerns of owners as well as true prevalence [40]. Based on a telephone survey, the most common disorders in dogs in the US were reported as musculoskeletal, dental, and gastrointestinal tract or hepatic disease [50]. Differences in the ranking of the most common disorders between these various data sources suggest that the information resource and data extraction methods can have a substantial impact on the results. Comparison between results from series of studies based on standardised core data collection and analysis methods is therefore more likely to offer safer inference between studies. This conclusion suggests the value of larger research programmes using cohort data collection nationally for more repeatable and comparable study results [13].

There are currently concerted efforts to identify key breed health issues by comparing disorder prevalence and risk between individual specific breeds and an appropriate comparator group such as crossbreds, other specific breeds [51] or all remaining dogs [52]. An application of the results from the current study would be to provide comparator disorder prevalence data on the wider dog population that could act as a baseline for the generation of breed-specific predispositions and protections. For comparative inference with the greatest reliability, breed specific data should be extracted from the same information resource using the same methods as the comparator group [52]. The findings from such comparisons can be used to develop breed specific health plans that prioritise common, severe or long duration disorders; an example of such breed health plans is shown by the UK Kennel Club’s ‘Breed health and conservation plan’ project [37]. Identification of differing predisposition and protection profiles for common disorders within and between breeds can promote improved understanding of the wider impacts from morphological and behavioural diversity selectively introduced into modern dog breeds. Awareness of these predispositions offers the prospect that co-ordinated health activities at a breed level may mitigate some of these negative welfare impacts [9, 53]. However, it is worth noting that direct comparison of prevalence values between breeds risks falling into the trap of univariable comparisons that do not account for confounding effects from other variables that may be associated with the disorder risk and that may differ between the breeds (e.g. age, sex, neuter status) [54]. The nature of such confounding is explored later in this discussion.

Strategies to improve canine health can be focused on dogs overall to reduce the welfare impact of common disorders such as obesity [55] or can be targeted to the specific needs of individual breeds [37]. The diversity of canine breeds, each with its own breed club structure that holds their own unique list of perceived priorities, makes the breed-focused approach understandable. And, indeed, this breed-focused approach is still a major plank of current health initiatives in dogs [37]. However, this breed-centric approach often tends to prioritise mitigation efforts on disorders that are either predisposed or perceived to have high genetic components within a breed above those that are common and modifiable, especially within the lifetime of the affected animals [12, 36, 56–58]. Especially for disorders that have high severity and duration, greater welfare gains may result from even modest reductions in the frequency of common disorders compared with even large proportional reductions in rare disorders [12, 36]. Although the current paper does not report on disorder risks within individual breeds, the current results could be useful as a baseline on general health issues in dogs to reframe future health reforms towards mitigation of more common disorders.

Veterinary expertise in managing and preventing common disorders offers many welfare benefits for dogs but it has always been challenging to prioritise allocation of teaching time across the breadth of topics needed within veterinary undergraduate curricula [59]. The findings of the current study enable visualisation of the majority component of a veterinarian’s daily workload and could assist in refining undergraduate veterinary teaching curricula to better equip new graduates with the necessary breadth of day-one skills [60–62]. Assuming that optimal allocation of undergraduate teaching times should take consideration of overall disorder prevalence, it is possible that the teaching time allocated for common disorders may currently be disproportionately short and that student education on the clinical management of these common disorders may rely excessively on the hidden curriculum or on experiences gained during extramural studies [63–66]. There is a risk that this scatter-gun approach may generate new graduates with widely varying beliefs about what constitures ‘best practice’, while also potentially allowing some important clinical topics to fall through the educational net with inadequate coverage. Similarly, the current findings can provide a picture of the typical clinical workload in primary-care practice for persons considering entering the veterinary profession or even for veterinary undergraduates contemplating the directionality of their future careers [67].

Access to reliable overviews of the main disorders managed in primary care practice can assist logistical exercises aimed at ensuring adequate resources for veterinary care at a national level. For example, the World Small Animal Veterinary Association has recently published a “List of Essential Medicines for Cats and Dogs” [68]. The list aims to support veterinarians in providing acceptable preventive care and treatment for the most frequent and important diseases in dogs and cats by facilitating medicines availability, drug quality, use and pharmacovigilance. Essential medicines were selected based on consideration of disease prevalence along with other factors including public/animal health relevance, clinical efficacy and safety, and comparative costs and cost-effectiveness. The value of such logistic exercises that require access to overall disease prevalence data has been especially evident at a national level during the recent UK plans to leave the European Union [69] and at an international level during the Covid19 pandemic [70]. Generation of ongoing and more detailed datasets describing the overall disease burdens in companion animal species looks set to become an increasingly valuable activity over the coming years as countries becomes ever more influenced by activities and events outside their own borders [71, 72].

Sample size estimation (power calculation) is a critical design component for any research project [73]. Failure to consider this step could lead to inclusion of too few animals with an under-powered study that misses significant differences that truly exist in the target population or, conversely, inclusion of too many animals can lead to wastage of resources and to ethical issues [74]. Paradoxically, a major challenge to sample size estimation for many studies in dogs has been access to reliable population-based prevalence data. This may partially explain the personal experience of the authors over many years that a large proportion of otherwise good epidemiological research has been published without an accompanying *a priori* sample size estimation and therefore offered limited inference, especially when negative findings are reported. As an additional support to improve future epidemiological study design for disorder studies in dogs, the current study provides generalisable results on disorder prevalence from a large cohort of dogs under UK primary care that can be used as the basis for sample size estimation (power calculation) [75].

Confounding describes the “mixing of effects” wherein effects from an exposure of interest (e.g. breed) on an outcome (e.g. a disorder) are conflated with the effects of another factor (e.g. age) that distorts the true relationship between the exposure and the outcome [76, 77]. Confounders may mask a true association so that it is missed or, conversely, generate an apparent association between the exposure and outcome even when no real association between them exists [78]. There are several approaches to dealing with the confounding problem, including randomisation, exclusion, matching and by using appropriate analysis [76]. Although randomisation reduces the necessity for prior consideration of potential confounding, the other three methods are reliant on *a prior*i causal consideration of both measurable and unmeasurable variables as potential confounders [78, 79].

The current study placed special focus on exploring associations between age, sex and neuter status with common disorders in an effort to assist future research to better understand and interpret these variables as potential confounders. The current results suggest important confounding effects for age, sex and neuter status in many common disorders of dogs. Among the 70 most common precise-level disorders, male dogs had increased odds for traumatic injury, ear disorder, brain disorder, musculoskeletal disorder, behaviour disorder, foreign body, upper respiratory tract disorder, and adverse reaction to drug. Conversely, bitches had higher odds for urinary system disorder, incontinence and claw/nail disorder. Some of these sex associations have previously been identified whereas the current study presents novel findings for others. It is worth noting that these latter novel findings should be treated with caution until supported or refuted by future confirmatory analyses. Disorders with previously reported associations with sex include otitis externa, aggression and seizure disorder in males [80–82] and urinary tract infection, mammary mass, urinary incontinence and cruciate disease in females [42, 83–86].

To add further complexity to the effects of sex on disorder occurrence, the current study also identified associations between neuter status and several disorders. Neutered animals had increased odds for obesity, dental disorder, musculoskeletal disorder, incontinence and behaviour disorder. Conversely, entire animals had higher odds for female reproductive disorder, male reproductive system disorder, underweight and parasite infestation. Some of these associations have been previously reported, such as obesity, osteoarthritis and urinary incontinence in neutered dogs [87–89]. However, it is often stated that ‘association does not imply causation’ and the dangers of making this leap from association to assuming causality are especially valid when trying to interpret the neuter status associations reported in the current paper [90]. Neuter status is a time-varying variable, with all dogs starting life as entire. Neutering itself is generally irreversible so that the proportion of neutered dogs rises with age, assuming neutering does not shorten life. Associations between neuter status and disorder therefore become heavily confounded by age. For example, although neutered dogs had a much higher prevalence of periodontal disease compared to entire dogs (17.34 % versus 8.56 % respectively), the median age of dogs with periodontal disease was quite old at 7.54 years. Since these periodontal disorder cases were, on average, older, they had more time to be neutered, which might account in part for this association. In addition, the current study was a cross sectional analysis with neuter status recorded at the date of final available record and therefore it is unknown whether the disorder preceded or followed the neutering event for each individual dog. To fully explore the effects of neutering on disease risk, cohort study designs are needed whereby each dog is followed over time from puppyhood, taking note of the dates of neutering and disease occurrence [76]. However, to date, such cohort studies have been rare in veterinary studies on companion animals due to their complexity as well as temporal and financial costs, especially for disorders that may occur many years after the neutering event [89].

The current study reported the median age of affected dogs at the end of 2016 for each of the common disorders. These values allow assessment of the typical age profile for dogs affected with these disorders. It is noticeable that disorders diagnosed in dogs aged over 9 years largely include degenerative and neoplastic disorders, such as osteoarthritis, heart murmur, skin mass and lipoma, whilst disorders diagnosed in dogs under six years largely include disorders associated with infection, allergy, behaviour, trauma or dietary indiscretion such as otitis externa, pyoderma, vomiting/diarrhoea, conjunctivitis and claw injury. To date, there has been limited published evidence on the age profiles of dogs affected with common disorders. However, the current findings are in line with a Korean report in which young dogs (< 1 year) most commonly presented for preventive medicine, diarrhoea, vomiting and infectious diseases while older dogs (over 10 years) presented more commonly with heart disease, kidney disease, Cushing’s disease, and mammary tumours [39]. Similar to the confounding effects described above, the results of the current study suggest that age should routinely be considered as a potential confounder in risk factor analyses for common disorders. For example, the median age in the UK varies widely between common dog breeds: French Bulldog 1.3 years [28], Bulldog 2.3 years [25], Chihuahua 2.8 years [27], Pug 3.0 years [33], Miniature Schnauzer 3.8 years [32], Rottweiler 4.5 years [34], German Shepherd Dog 4.7 years [29] and West Highland White Terrier 7.8 years [35]. A direct comparison of disorder profiles or prevalence between these breeds that did not account for age could spuriously suggest predispositions to typical disorders of young dogs in breeds such as the French Bulldog and Bulldog and predispositions to disorders of older dogs in breeds such as German Shepherd Dog and West Highland White Terrier. The current study highlights the complexity that is introduced into disorder occurrence from their associations with age, sex and neuter status and emphasises that careful consideration should be given to confounding variables when planning, designing and interpreting disorder studies in dogs [91].

Application of veterinary clinical data for epidemiological analyses have some important limitations that have been previously reported [13, 27, 42, 43]. Additional limitations for the current study included a tendency of veterinarians to commonly record presenting signs *in lieu* of formal biomedical diagnostic terms e.g. heart murmur rather than degenerative mitral valve disease, which prevents allocation of these true underlying diagnoses to the relevant specific disorders. Whilst this might underestimate the prevalence of specific disorders, the current results do reflect current primary-care practice more accurately. The process of disorder diagnosis and management in primary care practice is complex, with multiple factors such as client finances and expectations affecting the perceived need or importance for gaining full or precise diagnoses. These factors will therefore also impact the results reported in the current paper which should be interpreted as the prevalence of disorders diagnosed as opposed to the true prevalence of the underlying disorders in this population of dogs. The disorder prevalence values reported in the current study reflect the diagnosis levels in the current population of dogs. However, differing confounding effects from age, sex, neuter and other profiles in alternative populations could result in different prevalence values in these other populations that share the same inherent disorder propensities. The current study shows results from an array of association tests with the aim of detecting patterns of overall association. The provision of p-values may assist the reader to interpret these statistical results but it should be noted that these p-values did not include adjustments to account for multiple testing such as the Bonferroni correction [92]. Consequently, this elevates the possibility of Type I error (false positive findings) such that individual test results should be interpreted with caution [93–95]. Associations between sex and neuter with the individual disorders from the current study should be treated as exploratory, and as useful hypothesis generators, rather than as confirmatory. The current study reports disorder prevalence (i.e. the proportion of dogs with the disorder of interest during 2016) rather than incidence (i.e. the proportion of dogs that developed the disorder of interest for the first time during 2016). Prevalence studies are biased towards reporting disorders with longer duration (e.g. generally chronic conditions such as osteoarthritis, obesity) compared with disorders of short duration (e.g. generally acute conditions such as conjunctivitis, otitis externa) [36]. The study extracted the first listed presenting sign for disorders that were not ascribed a formal biomedical diagnosis. This term was reported in the precise-level diagnosis results. Given that many underlying pathologies present with multiple common presenting signs (e.g. gastroenteritis may present with vomiting, diarrhoea or malaise), this approach may have segregated these underlying pathologies across several terms at the precise-diagnosis level. The current study aimed to overcome this limitation by merging these terms into higher levels of abstraction in the grouped-level diagnoses.

## Conclusions

 This study of over 22,000 dogs under primary veterinary care builds on previous work within VetCompass and identifies the most commonly diagnosed disorders at a precise-level as periodontal disease, otitis externa, obesity, overgrown nail(s) and anal sac impaction. The most common disorder groups were dental, skin disorder, enteropathy, musculoskeletal and ear disorder. Sex and neuter status were commonly associated with disorder occurrence. The median age of affected dogs varied widely and showed patterns of associations with differing pathophysiological processes. The findings on the prevalence of the most common disorders can assist owners and veterinarians to prioritise preventive care and can enhance diagnosis of common disorders within dogs, and especially with an evidence-based differential focus within differing age and sex categories. In addition, the sex, neuter and age-specific differences noted between common disorders can assist future studies to improve their epidemiological study design to ensure higher validity of their results. Disorder occurrence in dogs is concluded to be highly complex but the patterns identified in the current study suggest that there are underlying rules that can offer owners, veterinarians and welfare scientists opportunities to better understand and control canine health.

## Methods

The data collection and analysis methods applied in the current study are similar to those recently reported to investigate disease risk in brachycephalic dogs [96]. The current study included dogs receiving primary veterinary care at practices sharing data with the VetCompass Programme in 2016. Dogs receiving veterinary care had either a) ≥ 1 electronic patient record (EPR) (VeNom diagnosis term, free-text clinical note, treatment or bodyweight) recorded in 2016 or b) ≥ 1 EPR recorded during 2015 and 2017. VetCompass shares anonymised EPR data with UK primary-care veterinary practices for welfare research [97]. Information available for analysis includes an animal and veterinary clinic identifier, date of birth, species, breed, sex and neuter, and also clinical information from free-text clinical notes, summary diagnosis terms [98] and treatment along with dates.

The study used a cohort design to estimate a one-year period prevalence (2016) for the most commonly diagnosed disorders [99]. Power calculation estimated 18,440 dogs were needed to report prevalence for a disorder diagnosed in 2.0 % of dogs with 0.2 % margin of error to a 95 % confidence level from a population of 905,544 dogs [100]. Ethics approval was obtained from the Royal Veterinary College Ethics and Welfare Committee (reference number SR2018-1652). All methods were performed in accordance with the relevant guidelines and regulations. Consent for use of the clinical data of the study dogs was obtained from the participating clinics and the animal owners.

The analysis included 22,333 dogs randomly sampled from the sampling frame of dogs under veterinary care in 2016. Following random ordering, the EPRs relating to 2016 were reviewed manually for all dogs to extract the most definitive diagnosis terms recorded for all disorders recorded during 2016 [101]. The manual review process was conducted under the direct supervision of the lead author (DON) by nine final-year veterinary undergraduate students. Disorder events in the cohort data were followed over time to determine the most definitive diagnosis term. Prophylactic (e.g. vaccination) or elective (e.g. neutering) interventions were excluded. Both pre-existing and incident disorder presentations were included. Disorders that were not recorded with a formal biomedical diagnostic term (e.g. ‘lameness’ or ‘lameness and hopping’) were extracted using the first presenting sign term (e.g. ‘lameness’). All diagnosis terms from the study were mapped to two levels of abstraction for diagnostic precision: precise-level precision and grouped-level precision as previously described [101]. Briefly, precise-level precision terms described extracted terms to the maximal diagnostic precision recorded within the EPR (e.g. *periodontal disease* would remain as *periodontal disease*). Grouped-level precision terms mapped the extracted diagnosis terms to a general level of diagnostic precision (e.g. *periodontal disease* would map to *dental disorder*).

Data cleaning and checking for internal validity was carried out in Excel (Microsoft Office Excel 2013, Microsoft Corp.). Statistical analysis used Stata Version 13 (Stata Corporation). Age (years) was defined at December 31, 2016 (latest date for dogs to be classified for each disorder). The one-year period prevalence values described the percentage probability of diagnosis at least once during 2016. Estimates for the 95 % confidence intervals (CI) were derived from standard errors based on approximation to the binomial distribution [102]. For each disorder, the median age (years) for cases was reported. Period prevalence was reported overall and also distinctly for males and females. For univariable analysis, categorical variables were compared using the chi-squared test and continuous variables were compared using the Mann-Whitney U test. Correlation between continuous variables was assessed using Spearman’s rank correlation coefficient [102]. Statistical significance was set at *P* < 0,0.05.

Random effects multivariable binary logistic regression modelling was used to estimate the relative odds for males versus females and neutered dogs versus entire dogs. A separate model was built for each disorder that included a standard bank of covariables to account for confounding (sex, neuter, breed, *bodyweight relative to breed/sex mean, age category* and *insurance*). The clinic attended was included as a random effect [76]. The authors applied an ‘information theory’ approach to decide on which covariables to include in these standard models [103]. Results for only sex and neuter status that were of *a priori* interest were reported from each regression model.

## Data Availability

The dataset supporting the conclusions of this article is available in the RVC Research Online repository http://researchonline.rvc.ac.uk/id/eprint/12714 .

## Abbreviations

CI: Confidence interval
EPR: Electronic patient record
IQR: Interquartile range
KC: The Kennel Club
OR: Odds ratio
